# Virus Shedding and Diarrhea: A Review of Human Norovirus Genogroup II Infection in Gnotobiotic Pigs

**DOI:** 10.3390/v16091432

**Published:** 2024-09-07

**Authors:** Charlotte Nyblade, Lijuan Yuan

**Affiliations:** Department of Biomedical Sciences and Pathobiology, Virginia Polytechnic and State University, Blacksburg, VA 24061, USA; charlottejn@vt.edu

**Keywords:** human norovirus, gnotobiotic pigs

## Abstract

For nearly twenty years, gnotobiotic (Gn) pigs have been used as a model of human norovirus (HuNoV) infection and disease. Unique in their ability to develop diarrhea and shed virus post oral challenge, Gn pigs have since been used to evaluate the infectivity of several genogroup II HuNoV strains. Nearly all major pandemic GII.4 variants have been tested in Gn pigs, with varying rates of infectivity. Some induce an asymptomatic state despite being shed in large quantities in stool, and others induce high incidence of both diarrhea and virus shedding. Non-GII.4 strains, including GII.12 and GII.6, have also been evaluated in Gn pigs. Again, rates of diarrhea and virus shedding tend to vary between studies. Several factors may influence these findings, including age, dosage, biological host factors, or bacterial presence. The impact of these factors is nuanced and requires further evaluation to elucidate the exact mechanisms behind increases or decreases in infection rates. Regardless, the value of Gn pig models in HuNoV research cannot be understated, and the model will surely continue to contribute to the field in years to come.

## 1. Introduction

Human norovirus (HuNoV) is one of ten viruses in the *Caliciviridae* family [1]. A small, positive sense, single-stranded RNA virus, HuNoV is the leading cause of food-borne gastroenteritis [1]. Spread via the fecal–oral route of infection, HuNoV causes gastrointestinal related symptoms, including nausea, diarrhea, and vomiting, in affected individuals [1]. All age groups are susceptible to infection, and repeat infections are common due to strain diversity and lack of long-lasting immunity [1]. Despite being discovered over half century ago in 1972, several key questions remain about HuNoV, including the cellular receptor, means of entry, and correlates of protection [1,2].

Part of the difficulty of elucidating HuNoV virology has been due to the finicky nature of cultivating it in vitro and in vivo [3]. Until 2016, no robust, replicable system of cell culture for HuNoV had been generated. The advent of human intestinal enteroids (HIEs) was a major breakthrough and has since been used to aid in neutralization assays, infection dynamics, and disinfection measures [3,4]. HIEs are an invaluable system; however, there remains a need for a robust in vivo model of HuNoV infection and disease in order to fully characterize the virus. Several lab animals have been tested for their ability to serve as a model of HuNoV infection and disease, including conventional and genetically modified mice, monkeys, gnotobiotic (Gn) calves, and Gn pigs [5,6,7,8,9,10]. However, only Gn pigs and calves fully recapitulate all aspects of HuNoV infection and disease. These animals are susceptible via oral challenge, develop diarrhea, shed virus, and seroconvert [9,10]. Between Gn pigs and calves, pigs have a more human-like gastrointestinal physiology. This, in combination with litter and body size, gives them an advantage over calves.

Gn pigs are derived via hysterectomy from near-term pregnant sows and maintained in sterile isolators throughout studies [11]. Due to the nature of derivation and the housing units, the piglets possess no microbiota [11]. The piglets also lack maternal antibodies since the thick placenta prevents the transfer of antibodies [11]. Gn pigs are immunologically naïve, but they are fully immunocompetent [11]. While Gn pigs are useful models for many aspects of HuNoV infection, including immune responses and pathogenesis, here, we aim to specifically review the rates and duration of clinical signs of infection (diarrhea and fecal virus shedding) following challenge with GII strains. Key parameters used to evaluate the effectiveness of HuNoV vaccines and therapeutics in Gn pig models include the incidence (percentage or rate), onset day, duration, cumulative fecal score, and area under the curve (AUC) (takes into account both the duration and the severity) of diarrhea. Virus shedding parameters include incidence, onset day, duration, peak fecal titers, and AUC of virus shedding. Protective efficacy against diarrhea or virus shedding is calculated as (1 − [% vaccinated pigs in each group with diarrhea or virus shedding/% mock-vaccinated control pigs with diarrhea or virus shedding]) × 100. Comparing these parameters among vaccine and control groups can characterize the effectiveness of a vaccine and create a predication of how well a vaccine may perform in clinical trials. Protection from diarrhea symptoms is the primary goal for all vaccines against viral gastroenteritis and is an easy clinical sign to monitor. Vaccines that are effective in reducing diarrhea severity in Gn pigs are likely to alleviate the most common symptoms of HuNoV gastroenteritis in humans. A vaccine that strongly reduces virus shedding titers and shortens the shedding duration suggests the vaccine would be effective at reducing interpersonal fecal–oral transmission.

## 2. Virus Shedding and Diarrhea Rates in Gn Pigs

A complete list of the virus shedding and diarrhea rates and duration in various GII HuNoV-inoculated Gn pigs can be found in Table 1. In studies where some groups of pigs received an additional treatment in combination with HuNoV, the summary table reports only infectivity rates of the control pigs challenged with HuNoV alone. Greater details for each virus strain can be found in the subsections below.

### 2.1. Infection with Pandemic GII.4 Strains of HuNoV

#### 2.1.1. GII.4 Bristol

One of the earliest Gn pig models of HuNoV infection to be reported used the strain HS66 (GII.4 Bristol) [10]. HS66 (GenBank accession No. EU105469) was isolated from the stool sample of a child who presented with diarrhea at Children’s Hospital in Columbus, OH [10]. In total, 73% of Gn pigs (*n* = 11) orally challenged with 2.5 × 10^6^ genome equivalents from the original stool sample shed virus in feces [10]. The average duration of virus shedding was three days when quantified by RT-qPCR, though shedding up to eight days was detected in some pigs when amplicon hybridization was used [10]. A total of 64% of the challenged pigs developed diarrhea after two days on average [10]. The diarrhea was typically mild, and the mean duration was two days [10]. These findings indicated for the first time that Gn pigs were not only susceptible to GII.4 HuNoV infection, but capable of displaying the range of clinical presentations seen in HuNoV cases.

HuNoV-positive intestinal contents from the infected pigs were collected, pooled, and used to inoculate a second group of Gn pigs (*n* = 7) to evaluate if HS66 could be serially passaged. Upon inoculation, the diarrhea rate increased to 86% [10,12]. The mean onset day remained the same (two days post inoculation), and the mean duration increased up to three days [10,12]. However, the opposite trend was observed in the virus shedding data, where there was a significant decrease in the number of pigs with virus in feces. In total, 29% of pigs had RT-PCR-positive fecal samples, and the duration of virus shedding decreased from three days on average to two [10,12]. The process of collecting HuNoV-positive intestinal contents was repeated, and a third set of Gn pigs (*n* = 4) were inoculated with HuNoV. During this third passaging, the virus-shedding rate recovered slightly (up to 50%), but was still lower than infection with the original human stool sample [10,12]. The mean duration of virus shedding remained at two days [10,12]. For diarrhea, the percentage rate remained higher than from the initial infection (75% versus 64%), but the mean duration dropped to one day [10,12]. When all passages were taken into consideration, HS66 induced a 55% virus-shedding rate and a 73% diarrhea rate [10]. The change in infectivity seen during viral passaging could be due to viral factors, host factors, or a combination of both. The initial viral inoculum was from a human stool sample, and therefore would likely need a few passages to adapt to pigs. Cheetham et al. speculated that a new clone in the HuNoV quasispecies could slowly become dominant in the pigs; however, more than two passages would be necessary to adequately amplify the clone [10]. Age and immune system maturity could also play a role in passaging effectiveness. Older animals with developed immune systems would be more likely to mount a robust innate anti-viral response compared to a younger animal.

A second study performed with HS66 reported higher infectivity rates. Here, a higher dose of HS66 (5.4 × 10^6^ genome equivalents) was used to challenge neonatal Gn pigs (*n* = 32) [13]. In this study, 91% of pigs shed virus in feces [13]. The mean duration of shedding was greater than before, up to four days on average [13]. In total, 84% of pigs developed diarrhea [13]. The mean duration of diarrhea was also increased up to four days [13]. The severity of diarrhea was not reported for this study.

HS66 was also used for vaccine challenge studies. In one such study, 33-day-old Gn pigs were orally challenged with 5.4 × 10^6^ genomic equivalents of the virus [14]. The control pigs (*n* = 7), which received adjuvants but no HuNoV vaccine antigen prior to challenge, developed diarrhea and shed virus. A total of 57% of pigs (4/7) shed virus and 100% (7/7) developed diarrhea [14]. Diarrhea was mild, as indicated by the mean cumulative score of 10. The peak fecal titers and duration of diarrhea were not reported in this study. Collectively, these studies indicate that Gn pigs are a valid model of HuNoV GII.4 HS66 infection and disease.

#### 2.1.2. GII.4 Farmington Hills

The Farmington Hills variant was the next pandemic GII.4 strain to arise after GII.4 Bristol in 2002 [22]. In order to evaluate if GII.4 Farmington Hills was also infectious in Gn pigs, a dose–response study was conducted. The Farmington Hills variant Cin2 (GenBank accession No. JQ965810) was isolated from a volunteer enrolled in a HuNoV vaccine study at Cincinnati Children’s Hospital [15]. At 33–34 days of age, Gn pigs (*n* = 3–6) were orally challenged with one of seven doses of Cin2 and monitored for seven days post challenge. Dosages were as low as 10^2^ RNA copies and went up to 10^6^ RNA copies. As in the case of HS66, Cin2 was found to be infectious to varying degrees in Gn pigs. At the lowest tested dose, only 25% of the pigs shed virus and none developed diarrhea [15]. The next two doses (3.2 × 10^3^ and 2 × 10^4^) both resulted in 67% of pigs shedding virus [15]. Only one pig in the 10^3^ dose group developed diarrhea [15]. The remaining tested doses all resulted in 100% virus shedding [15]. Increased doses also resulted in increased diarrhea rates, as all but one of the final four doses caused 100% diarrhea [15]. The only high-dose group to not reach 100% diarrhea was the 4 × 10^5^ group, which had 75% diarrhea [15]. The duration of virus shedding was around 1–3 days across groups, though a more prolonged duration of 6.3 days was observed in the 2 × 10^5^ group [15]. The duration of diarrhea was as low as 1 day and as high as 5 days [15]. This is the most extensive dose–response study in Gn pigs that determined the median infectious dose (ID_50_) and median diarrhea dose (DD_50_) of a GII.4 HuNoV variant. The ID_50_ of Cin2 (~2.5 × 10^3^) in Gn pigs closely mimics that observed in human volunteers (2.8 × 10^3^) [15]. Several methods were used to calculate the ID_50_ and DD_50_, including Reed–Muench, Dragstedt–Behrens, Spearman–Karber, Logistic Regression, Exponential, and Approximate Beta-Poisson models [15]. Details of individual statistical methods can be found in this paper. All methods used placed the ID_50_ within 10^3^ genomic copies and the DD_50_ within 10^4^ genomic copies. A summary of all the ID_50_ and DD_50_ results can be found in Table 2.

#### 2.1.3. GII.4 Den Haag

Following GII.4 Farmington Hills, GII.4 Den Haag was found, a pandemic variant isolated from the Netherlands in 2006 [22]. Several studies have been conducted with two Den Haag variants over the years. Two of the earliest are briefly discussed below.

The viral inoculum was isolated from the stool sample of a child with HuNoV gastroenteritis at Cincinnati Children’s Hospital [16]. Several doses of the GII.4 Den Haag (GenBank accession No. KC990829) variant named GII.4/2006b, from 10^3^ to 10^6^, were tested in neonatal (*n* = 3 or 6) and month-old pigs (*n* = 3–5). Virus shedding ranged from 50–100% in neonates, with a higher dose correlating with an increased shedding percentage [16]. All pigs inoculated with the low dose shed virus for one day; pigs infected with a higher dose shed virus for three to four days [16]. In month-old animals, virus shedding ranged from 25 to 100% [16]. Again, higher doses resulted in higher shedding percentages and prolonged shedding durations. Based on this study, the GII.4 Den Haag variant is less infectious than the GII.4 Farmington Hill variant in Gn pigs, as evidenced by the higher ID_50_ (6.43 × 10^4^) of Cin2 in month-old pigs (Table 2) [16]. The ID_50_ was calculated individually for neonates and month-old pigs. For both groups, the Reed–Muench method was used [16]. The proportionate distance between dilutions above and below the 50% endpoint was calculated using the following formula:

(1) $$Propotionate\;distance\;(PD)=\frac{Percentage\;infected\;at\;dilution\;next\;about\;50\%-50\%}{Percentage\;infected\;next\;about\;50\%-Percentage\;infected\;at\;dilution\;next\;below\;50\%}$$

Pigs were considered infected if a rectal swab or intestinal content samples was positive by conventional RT-PCR or real-time RT-PCR. After calculating the proportionate distance, the ID_50_ was calculated using the following formula.

(2) log50% end point=(log dilution above 50%)−(PD×log dilution factor)

A separate study was conducted using another GII.4 Den Haag (GenBank accession No. GU325839) variant named HS194, with a higher inoculum dose. Briefly, 11–13-day-old pigs (*n* = 10) were orally challenged with either 2.4 × 10^9^ or 3 × 10^10^ genomic equivalents of virus isolated from the stool of a child with watery diarrhea [17]. The onset day of fecal virus shedding was 4.8 days post inoculation [17]. The mean duration of virus shedding was much greater than that observed in the previous GII.4/2006b study, continuing for 8.8 days on average [17]. Peak titers were in the 10^4^ genomic equivalents/mL range [17]. Interestingly, no diarrhea was observed in any of the HuNoV HS194 inoculated pigs despite the high-challenge dose and high titers of fecal virus shedding. Based on this study, HS194 is limited in its utility for future vaccine studies. The lack of diarrhea is a concern, as there is no way to determine if vaccines will prevent symptoms of infection. Additionally, the high-challenge dose is disproportionate to the ID_50_ seen in human studies.

#### 2.1.4. GII.4 New Orleans

GII.4 New Orleans emerged as the next pandemic strain in 2009 to overtake GII.4 Den Haag [22]. Only one study thus far has reported the infectivity of GII.4 New Orleans in Gn pigs. At 8–14 days of age, pigs (*n* = 14) were orally challenged with 9.1 log_10_ (1.26 × 10^9^) genome equivalents of the GII.4 New Orleans strain HS292 (GenBank accession No. KJ407073) [18,23]. In this study, the infected pigs did shed virus; however, the overall percentage was not reported. The mean onset day was 2.8, with a mean duration of shedding lasting 11.6 days [18]. The infected pigs were shedding high titers of virus as well, with mean peak titers reaching 6.2 log_10_ (1.58 × 10^6^) genome equivalents/mL [18]. No numerical values were reported for diarrhea data. The high, prolonged fecal titers make the GII.4 New Orleans model promising; however, as in the case of HS194, diarrhea data are needed to bolster the model’s utility.

#### 2.1.5. GII.4 Sydney

Unlike previous pandemic strains that were typically replaced by a new variant every few years, GII.4 Sydney has remained dominant since its emergence in 2012 [22]. While a formal Gn pig model of GII.4 Sydney has yet to be published, pilot studies are ongoing.

GII.4 Sydney (GenBank accession No. PQ046323) was isolated from an adult human stool sample and used to inoculate Gn pigs (*n* = 11) in our laboratory at a dose of ~10^5^ RNA copies. Out of 11 inoculated pigs, 7 shed virus (81%). The mean duration of shedding was 3 days, and the mean peak titers were 8.22 × 10^3^ genome copies/mL. The incidence of diarrhea among pigs was lower than the virus-shedding rate, at 64% (7/11) pigs. The mean duration of diarrhea was similar to the virus shedding, at 3.85 days on average (unpublished data). While preliminary data suggest that Gn pigs are a good model for GII.4 Sydney, further work fine-tuning the model is desirable. For example, GII.4 Sydney is hypothesized to target different HBGAs and have an improved affinity for host cell receptors [1]. GII.4 Sydney is also hypothesized to evade herd immunity [1]. Determining if the virus maintains these qualities in Gn pigs would provide support for Gn pigs as a robust HuNoV model. Additionally, it should be determined if the development of clinical signs is dosage-dependent in pigs and how age impacts susceptibility to GII.4 Sydney 2.1.6. KU13206.

KU13206 (GII.4) was isolated from a patient suffering from acute gastroenteritis (acquired before 2018; variant not reported), and a dose–response study was conducted in post-weaning Gn piglets [19]. At four weeks of age, pigs (*n* = 4/dose) were orally challenged with 1 × 10^5^, 1 × 10^6^, or 1 × 10^7^ genomic equivalent copies of the virus [19]. In total, 50% of pigs in the 10^5^ and 10^7^ challenge groups and 75% of pigs in the 10^6^ dose group shed virus in feces [19]. The duration and magnitude of virus shedding was not reported. Similar to the Cin2 dose–response study, diarrhea incidence occurred in a dose-dependent manner. No pigs inoculated with the lowest dose of KU13206 developed diarrhea [19]. Mild diarrhea was observed beginning at PID2 in one pig (25%) inoculated with 10^6^ genomic copies [19]. At the highest dose, 75% (3/4) pigs developed diarrhea; of these pigs, two developed severe watery diarrhea by PID2 and the third developed moderate diarrhea at PID3 [19]. These data suggest that the dose of virus used for inoculation is highly strain-specific and needs to be fine-tuned for each strain. Despite KU13026 also being a GII.4 strain, it appears to require a much higher dose of virus to induce diarrhea compared to Cin2 or 2006b.

### 2.2. Infection with Non-GII.4 Strains of HuNoV

While GII.4 strains are the dominant genotype isolated from outbreaks, they are not the only ones. Other strains that make up a large portion of outbreaks include GII.17, GI.5, GII.6, and GII.2 [24]. These data reflect the diversity of HuNoV and highlight the need for strain-specific animal models.

#### 2.2.1. GII.6

As of July 2024, GII.6 strains make up nearly 10% of typed strains isolated from HuNoV outbreaks in the United States [24]. Much like the testing of GII.4 Sydney in Gn pigs, work with GII.6 HuNoV is ongoing. A GII.6 strain (GenBank accession No. PP661666) isolated from an adult human fecal sample was found to be infectious and pathogenic in neonatal (*n* = 2) and month-old (*n* = 4) Gn pigs in our laboratory. Two neonates inoculated with 5 × 10^4^ genome copies shed virus for 8 and 9 days, with peak fecal titers reaching 10^5^ genome copies/g. Only one pig developed mild diarrhea that lasted for two days. In month-old pigs at a higher dose (10^6^ genome copies), 100% of pigs shed virus. The mean shedding duration was shorter than in the neonates (5.75 days); however, one pig did shed virus for 8 days. The diarrhea incidence was 100% amongst the older pigs and more severe, lasting for 8.5 days on average (unpublished data). As in the case of GII.4 Sydney infection, further work is warranted on this model. The small study n is a significant limitation; so, additional pigs are needed to establish the Gn pig GII.6 challenge model in both age groups.

#### 2.2.2. GII.12

GII.12 strains have been rising in predominance over the last few years. This genotype has been detected in several surveillance studies of pediatric patients, including studies in Thailand, Nicaragua, and South Africa [25,26,27].

The GII.12 strain HS206 (GenBank accession No. HQ664990) was isolated from a Korean patient with gastroenteritis; 9.5 log_10_ (3.16 × 10^9^) genomic equivalents were used to orally challenge 8–11-day-old pigs (*n* = 6) [20]. In total, 100% of the infected pigs shed virus in feces for an average duration of 7 days [20]. Peak fecal HuNoV titers averaged 6.0 log_10_ (1 × 10^6^) genome equivalents/mL [20]. The diarrhea rate was considerably lower than the virus-shedding rate in challenged pigs. A total of 33% of pigs developed mild diarrhea over the course of 21 days, with the mean duration being 1.8 days [20].

A recombinant GII.3[P12] strain (GenBank accession No. MN199033) was isolated from a Korean patient suffering from acute gastroenteritis and used for infection of 4-week-old Gn pigs [21]. Pigs (*n* = 7) were challenged with 10^7^ genome copies of the virus [21]. The authors did not determine diarrhea rates in the piglets; they only monitored fecal virus shedding. All challenged pigs shed HuNoV, with the onset day of shedding beginning between 1 and 2 days post inoculation [21]. Greater details on the clinical signs of infection were not reported, as the primary focus of this paper was intestinal immune responses post HuNoV infection.

### 2.3. Modeling Additional Components of GII Replication

In humans, the severity of GII HuNoV infection can be moderated by several factors. The impact of these factors can be modeled in Gn pigs. A summary of these findings is presented in Table 2 and greater details are provided in the following subsections.

#### 2.3.1. Sector Status and HuNoV Infection

Secretor status is a well-documented element that influences HuNoV infection [28]. Secretor status refers to the expression of histo-blood group antigens (HBGAs). HBGAs are carbohydrates expressed on mucosal surfaces, red blood cells, and in secretions like saliva [28]. Their expression is modulated by several genes, including *FUT2*, *FUT3*, and *ABO*. These genes are responsible for the addition of different sugars onto precursor glycans, resulting in the fully formed HBGA [28]. *FUT2* and *ABO* are responsible for the generation of H and A antigens, respectively, while *FUT3* controls Lewis antigen expression [28]. Because HuNoV utilizes HBGAs as a coreceptor, secretor-positive status is associated with an increased risk of infection [1,28].

Gn pigs that are secretor-positive are also at an increased risk [29]. This finding was determined by using archival tissue samples from a Gn pig infectivity study with the GII.4 Bristol strain HS66. The study had found previously that 44% of GII.4 HS66-inoculated pigs and 59% seroconverted shed virus in feces, leading to the hypothesis that H or A antigen expression influenced HuNoV infectivity [10,29]. HBGA typing was performed via immunofluorescent staining, and the A/H phenotypes were then matched back to virus shedding results. More pigs with the A+H+ phenotype shed virus compared to the A-H- phenotypes, 51% (29/57) versus 12.5% (1/8), respectively [29]. Additionally, the diarrhea rates in A+H+ pigs were significantly higher, reaching up to 81% (46/57) compared to the A-H- pigs at 37.5% (3/8) [29].

#### 2.3.2. Bacteria and HuNoV Infection

Another factor that has been shown to influence HuNoV infections is the presence of bacteria. Bacterial cofactors are essential for the cultivation of HuNoV in B cells [30,31,32]. The addition of *Enterobacter cloacae* (*E. cloacae*), which expresses H antigen, was able to enhance HuNoV replication in B cells, whereas the addition of non-HBGA-expressing *Escherichia coli* did not [32]. Enhanced infectivity has also been demonstrated in vivo; a study of South African infants found increased diarrheal risk in children with higher percentages of *E. cloacae* in their gut microbiome [33]. These observations have led to several studies evaluating if this phenomenon holds true in Gn pigs. The results have been mixed, suggesting there may be multiple mechanisms through which bacteria influence HuNoV infections. A summary of studies evaluating connections between bacteria and HuNoV can be found in Table 3.

Because *E. cloacae* was observed to increase HuNoV infection in B cells, this bacterium was one of the first to be evaluated in combination with HuNoV in Gn pigs. Gn pigs (*n* = 8) were pre-colonized with *E. cloacae* at 3, 4, and 5 days of age after which they were challenged with 2.74 × 10^4^ genome copies of a GII.4 Den Haag variant [35]. A separate set of Gn pigs (*n* = 9) were orally challenged with the same dose of HuNoV but remained bacteria free. Diarrhea rates were similar between the control and *E. cloacae*-colonized pigs. At post-inoculation day (PID) 3, 44% and 50% of pigs in the control and *E. cloacae* groups, respectively, had diarrhea [35]. By PID7 and 10, 100% of pigs in both groups had diarrhea [35]. A similar trend was observed in the virus-shedding rates, where 100% virus shedding in both groups was reached by PID7 [35]. However, *E. cloacae*-colonized pigs had significantly lower cumulative shedding titers relative to the controls [35]. The colonized pigs also had a significantly shorter mean duration of virus shedding at PID10 than the control (6.7 versus 5.5 days) [35]. This study indicated that *E. cloacae* colonization reduced HuNoV replication capacity in the intestine tissues of Gn pigs.

The opposite result was observed in a study utilizing Gn pigs transplanted with a human gut microbiota (HGM). A stool sample collected from a vaginally delivered, breastfed male infant was used to transplant an HGM into newborn Gn pigs (*n* = 11) [36]. Pigs were colonized with the sample for two days, and then challenged with the same GII.4 Den Haag strain used in the *E. cloacae* study. At the early stages of infection (PID1-3), HGM pigs had a lower diarrheal rate than controls (9% versus 58%) and higher virus-shedding rate (100% versus 84%) [36]. Through the remaining stages of infection (PID4-10), HGM pigs ended up with a higher diarrhea rate (100% versus 78%) [36]. Both groups had 100% virus-shedding rates between PID4-10 [36]. From PID4-10, HGM pigs had diarrhea for 3.8 days and control pigs had diarrhea for 2 days [36]. During this time, HGM pigs also shed virus for longer. The mean duration of virus shedding was 4.9 days for the control pigs and 6.8 days for the colonized pigs [36].

Supporting the results of the HGM transplantation study is a recent report of the impact of *Escherichia fergusonii* (*E. fergusonii*) colonization on GII.12 HuNoV infection. Like *E. cloacae*, *E. fergusonii* is a commensal bacterium expressing HBGAs [20]. Briefly, 6–9-day-old Gn pigs (*n* = 6) were inoculated with *E. fergusonii* for two days prior to challenge with 9.5log_10_ (3.16 × 10^9^) genome equivalents of GII.12 HuNoV HS206 [20]. Age-matched control pigs (*n* = 6) remained bacteria-free and received only HuNoV. *E. fergusonii* did not have a significant impact on the diarrhea rates of inoculated pigs, though the total percentage of colonized pigs with diarrhea was trending higher than that of controls (40% vs. 33%) [20]. There was also no significant difference in other diarrhea parameters. The fecal virus-shedding rate in *E. fergusonii*-colonized pigs was 83%, and 100% in control pigs [20]. Despite this, *E. fergusonii*-colonized pigs had higher cumulative HuNoV titers in feces during the middle and late study stages (PID6–14 and PID15–21) [20]. Colonized pigs also had a significantly longer duration of virus shedding (11.8 days) compared to the controls (7 days) [20].

While *E. fergusonii* boosted the virus shedding severity post GII.12 infection, a more limited increase in infectivity post colonization was seen with pilot studies of Cin2 and GII.4 Sydney in our lab (unpublished data). The GII.4 Sydney-challenged pigs (*n* = 2) who were pre-colonized with *E. fergusonii* shed virus for 1-2 days longer than the non-colonized control (*n* = 1). However, only one colonized pig had higher peak fecal virus titers. The *E. fergusonii*-colonized pigs challenged with GII.4 Sydney had diarrhea for one day longer than the non-colonized control and a higher mean cumulative diarrhea score. Colonized, Cin2-challenged pigs (*n* = 2) also had higher cumulative diarrhea scores, but no difference in other diarrhea parameters. Colonized Cin2-challenged pigs had higher peak fecal titers compared to the non-colonized control (*n* = 1). However, there was no difference in the virus shedding duration. Additionally, for all challenged pigs, the fecal virus titers were very low (10^2^ genomic copies/mL). It is important to note that these are results from pilot studies. While they hint at interesting connections between *E. fergusonii* and HuNoV, continued investigation is necessary before firm conclusions can be drawn. A 2019 study with *Lactococcus lactis* (*L. lactis*) also observed a minimal increase in virus shedding post bacterial colonization. Four-day-old Gn pigs (*n* = 3–6) were orally gavaged with 10^9^, 10^10^, or 10^12^ CFU of engineered *L. lactis* expressing HuNoV VP1 or wild-type *L. lactis* [34]. The pigs were challenged twenty days later with 10^7^ genomic RNA copies of HuNoV GII.4 strain 766. Virus shedding was monitored for five days post challenge. While there was no statistical significance between the groups at any time point, there was a slight trend of increased viral shedding seen with higher doses of *L. lactis* [34]. Pigs that received 10^9^ CFU of *L. lactis* had fecal HuNoV titers hovering around 3 log_10_ (1000) RNA copies/g throughout the study, whereas pigs treated with 10^12^ CFU had titers in the range of 4 log_10_ (1 × 10^4^) to 5 log_10_ (1 × 10^5^) RNA copies/g. From these studies, it is clear that bacteria play a role in the infectivity of HuNoV, but the mechanism is yet to be defined.

#### 2.3.3. Immunocompromised Health Status and HuNoV Infection

In immunocompetent individuals, symptoms of HuNoV typically self-resolve within two-three days, and virus shedding occurs for a median of four weeks post infection [1,39]. However, suppression of the immune system in humans is well associated with more severe infections and prolonged shedding [1,40]. Chronic HuNoV infections have been reported in transplant, cancer, and severe combined immunodeficiency (SCID) patients [40]. The ability to model this immunosuppression in animals has important implications. Animal models could be used to develop therapeutics that are safe in these patients while mitigating the more severe symptoms and long-term shedding. The models can also be used to evaluate viral evolution rates, as immunocompromised patients with chronic infections have higher rates of within-host evolution [1]. A summary of the studies evaluating HuNoV infection in immunocompromised Gn pigs is presented in Table 3.

The advent of CRISPR/Cas9 technology has allowed for the generation of Gn pigs with immune deficiencies, including T cell, B cell, and natural killer (NK) cell knockouts [38]. In SCID pigs that are T-B-NK-, oral challenge with GII.4 Den Haag results in an increased and prolonged HuNoV infection. Fecal virus shedding was detected up to 27 days in T-B-NK- pigs (*n* = 12) but only up to 16 days in wild-type (WT) Gn pigs (*n* = 13) [38]. Higher peak titers were also detected in PID1-3 and PID11-17 in the knockout pigs compared to the WT [38]. Despite the more prominent virus shedding in T-B-NK- pigs, there was no significant difference in their diarrhea incidence [38]. A similar study was performed a few years later, but this time evaluating GII.4 infection in Gn pigs with a different naturally occurring SCID phenotype. These pigs had no T or B cells, but intact NK cell populations (T-B-NK+) [18]. Pigs (*n* = 12) were challenged with a GII.4 New Orleans variant. As before, there was no significant difference in the incidence of diarrhea between SCID and WT pigs (*n* = 14) [18]. Unlike the total knockouts, T-B-NK+ pigs did not have more severe virus shedding compared to WT pigs. There was no statistical significance in cumulative fecal titers, duration of shedding, and peak titers between the groups [18].

Immunocompromised phenotypes can also be generated through medication. In humans, the use of cholesterol-reducing drugs (statins) can cause immunosuppression via the downregulation of IFNα and T cell activation pathways [16,17]. Other drugs known to inhibit the immune system include chemotherapeutics or immunosuppressants given prior to transplant surgeries, which explains the incidence of chronic HuNoV infections in cancer and transplant patients [40].

Three studies in Gn pigs have found that simvastatin treatment causes more severe HuNoV infections. All studies used GII.4 Den Haag strains for challenge. In the first study, simvastatin-treated pigs (*n* = 11) shed virus for 15.1 days on average compared to 8.8 days in non-treated pigs (*n* = 10) [17]. Simvastatin treatment also resulted in higher mean daily HuNoV titers [17]. No diarrhea was observed in non-treated pigs; comparatively, 100% of simvastatin-treated pigs developed diarrhea [17]. In the second study, simvastatin treatment resulted in higher virus-shedding rates at three HuNoV challenge doses (10^3^, 10^4^, and 10^5^) [16]. Mirroring the first study, the simvastatin-treated pigs (*n* = 4) shed virus for more days and had higher peak titers than the non-treated animals (*n* = 3–5) [16]. Diarrhea rates were also increased in the simvastatin-treated animals, with 79% of pigs developing diarrhea [16]. Comparatively, the non-treated control animals had diarrhea rates of 38% [16]. In the final study, Gn pigs were vaccinated with HuNoV P particles from a 1997 Farmington Hills variant or a placebo control. Additionally, a subset of pigs in each treatment group was treated with simvastatin for eleven days prior to virus challenge. The virus strain used for challenge was the 2006b variant. Vaccinated, non-simvastatin-treated pigs (*n* = 9) had a reduced incidence of diarrhea compared to their respective controls (*n* = 6) (83% versus 44%, respectively) [37]. However, vaccinated simvastatin-treated pigs (n = 6) did not experience the same reduction in diarrhea rates. In total, 100% of vaccinated simvastatin-treated pigs developed diarrhea versus 83% of the matched controls [41]. Vaccinated simvastatin-treated pigs had a slightly longer mean duration of diarrhea than controls (3 versus 2.5 days) and the same AUC score (8.8) [41]. There was no statistical difference in virus-shedding incidence between the vaccinated and control groups regardless of simvastatin treatment [37,41].

## 3. Discussion

The global burden of HuNoV is immense. Based on recent report from the WHO, 685 million diarrheal cases in all age groups are caused by norovirus [42], causing an estimated a total of USD 4.2 billion in direct healthcare costs and USD 60.3 billion in societal costs per year [43]. While research has advanced significantly since the virus’s discovery, several factors make HuNoV difficult to study. The host receptor protein for HuNoV is unknown. While CD300lf has been identified as the murine norovirus (MNV) receptor, this protein is not utilized by HuNoV [1,44]. Another factor making HuNoV difficult to study is the virus’s genetic diversity. This complicates vaccine development as immune responses appear to be largely variant-specific [1,45]. Furthermore, the genetic diversity complicates the culture of the virus in vitro. Some variants are amenable to culture in HIEs; however, others do not readily infect the mini guts [46].

The use of Gn pigs has helped to build our understanding of HuNoV. Since the 1960s, Gn pigs have been an incredibly versatile tool in biomedical research. Their immunologically naïve state, in combination with microbiota-free body, allows for the evaluation of pathogen-specific immune responses [47]. This physical state also ensures there are no contaminating bacteria or porcine pathogens. Gn pigs have similar gastrointestinal physiology to humans, which enhances the translational potential of these pigs [47]. The first reported Gn pig model of HuNoV was established in 2006 at the Ohio State University utilizing a pandemic GII.4 Bristol strain as inoculum. Since then, several other strains have been tested, cementing Gn pigs as pivotal players in the HuNoV field.

Gn pigs are the most human-like animal model that fully recapitulates all aspects of HuNoV infection. None of the lower-order species develop diarrhea after inoculation with HuNoV [5]. Adult mice infected with MNV shed virus; however, they do not develop diarrhea [5,48]. MNV-infected suckling mice develop diarrhea and shed virus [5]. However, MNV has a different cell tropism than HuNoV, which limits the translational potential of the suckling mouse model. MNV infects immune cells, whereas HuNoV targets intestinal enterocytes [1,49]. Additionally, MNV utilizes a different receptor than HuNoV [1]. A humanized mouse model of HuNoV infection has been developed, but this model also has significant limitations. First, viral replication was only supported by viral intraperitoneal injection; this is a non-natural route of HuNoV infection [48]. Additionally, the altered immune state of the mice (Rag^−/−^γc^−/−^) would confound immune responses induced during vaccine studies [48].

Non-human primates and Gn calves have also been evaluated as candidate models. Non-human primates will seroconvert and shed virus in feces [5,8]. However, much like adult mice, monkeys do not develop diarrhea [5,8]. The lack of diarrhea, in combination with care expenses, make monkeys not suitable for routine use as a HuNoV model. Gn calves are the only model besides Gn pigs that develops diarrhea, shed virus, and mount HuNoV specific immune responses [9]. Compared to Gn pigs, Gn calves have a gastrointestinal physiology less similar to that of humans. Calves have a four-part stomach, whereas pigs and humans have a singular compartment.

In humans, a typical HuNoV infection has an incubation period of 1–2 days, with symptoms lasting for 2–3 days, and viral shedding occurring for a median of 1 month post infection [1]. Gn pigs mimic this timeline well. Pigs typically shed virus and develop diarrhea within 1–3 days post inoculation. The duration of diarrhea is shorter than the duration of virus shedding. Viral genomes have been detected in feces up to 21 days post infection [20]. This highlights the ability of Gn pigs to replicate the prolonged virus shedding seen in human cases. The ID_50_ in Gn pigs is similar to the ID_50_ in humans. In healthy, secretor-positive adults, the ID_50_ of Norwalk virus is 1.3 × 10^3^ genome equivalents. [50] In month-old, A+/H+ pigs, the ID_50_ of Cin2 in Gn pigs is ~2 × 10^3^ genome equivalents [15]. Children and the elderly are both more likely to experience severe HuNoV infections, and Gn pigs replicate this trend as well. In neonatal pigs, the ID_50_ of 2006b is ≤2.74 × 10^3^ genome copies [16]. Comparatively, the ID_50_ for month-old pigs is 6.43 × 10^4^ genome copies [16]. The age-associated trend may be due to immune system maturity, though studies of serology and cellular immunity post challenge would be necessary to validate this hypothesis.

One limitation to the Gn pig model is that virus-shedding and diarrhea rates can vary significantly between studies. There is no one answer to the variation seen, and it may be due to a combination of factors. Dosage is one such factor. While 100% fecal shedding was observed in one study at a dose of 8 × 10^4^ genome copies, higher doses typically induced greater peak fecal titers and prolonged shedding. Diarrhea appears to be more dependent on dosage than virus shedding. However, there are exceptions to this as HS194 did not cause diarrhea in any pigs despite a dose of 10^10^ genome copies. Variation in symptom presentation can also be observed in human cases. An outbreak of HuNoV in US military trainees caused diarrhea in 67% of affected individuals [51]. Comparatively, an outbreak in a Taiwanese psychiatric facility had a higher diarrheal rate of 88% [51]. Asymptomatic carriage of HuNoV is also possible, though it occurs at much lower rates. A total of 1–3% of asymptomatic food handlers in South Korea shed norovirus in stool; in the UK, 16% of asymptomatic individuals had HuNoV-positive fecal samples [51]. Again, there is no one answer to the variation and it is likely a combination of host and innate viral factors.

Sometimes the variation in HuNoV infections in vivo can be traced back to specific factors like gut bacteria. The influence of bacteria on HuNoV is multifaceted. *E. fergusonii* and bacterial cocktails have been shown to increase HuNoV infectivity in vivo, while *E. cloacae* had the opposite effect. One of the leading hypotheses for the increased infectivity is that bacteria bind HuNoV and bring it in closer proximity to gut cells [30,32,52]. This theory is supported by immunofluorescent staining that shows colocalization of HuNoV particles and bacterial cells on the tips of intestinal villi [20,35]. However, this same colocalization supports the theory put forward by Lei et al. in their *E. cloacae* study that bacteria serve as a barrier to infection by blocking access to the intestine. Proximity is not the only reason proposed for bacteria’s ability to modulate HuNoV infectivity. Bacterial binding can stabilize virions, thereby increasing their transmission potential [52]. Some commensal bacteria promote an immune tolerant state, resulting in less hostile environments for enteric viruses to thrive in [52].

Gn pigs are also ideal for measuring how host factors, such as secretor status, impact viral replication. For both infants and adults, secretor status is an important predictor of HuNoV susceptibility. In a study of Nicaraguan infants, the incidence of GII infections was nearly four-fold higher in the secretor-positive children compared to those that were secretor-negative [53]. The incidence of GI infections, however, was similar between secretor groups [53]. A study of Norwalk virus infection in adult volunteers found 100% infection rate in secretor-positive individuals compared to 0% infection in those who were secretor-negative [50]. A similar study in adult volunteers was conducted using GII.4 as the challenge inoculum; again, secretor status was associated with significantly higher risk of infection. Here, 70% of secretor-positive individuals were infected, with the majority developing symptoms and having HuNoV-positive stool samples for several days [54]. Comparatively, only one non-secretor (6%) was infected. This individual was asymptomatic and only shed virus for a single day [54]. Thus far, only one study has been performed in pigs with the interactions of HBGAs and HuNoV being the primary focal point [29]. In all the studies of HuNoV infection and immunity in our laboratory, only H+/A+ Gn pigs were used. Cheetham et al.’s 2007 study was performed retroactively with archived tissues, finding decreased infectivity rates in secretor-negative compared to secretor-positive pigs [29]. The study also determined the ability of different GI and GII VLPs to bind to HBGAs in the tissue sections. GI.1, GII.1, GII.3, and GII.4 VLPs all bound to A+ or H+ duodenal tissues, with extensive binding found for GI.1 and GII.4 particles [29]. GII.1 and GII.3 VLPs were less widely distributed in the tissues [29]. The diversity in binding patterns reflects the data seen in human challenge studies, suggesting Gn pigs are ideal for evaluating connections between secretor status, infectivity rate, and symptom severity. Moving forward, in vivo studies with newer pandemic and non-pandemic GII variants can help boost our understanding of strain-specific HBGA binding preferences.

## 4. Conclusions

From modeling the earliest pandemic GII.4 variant to current circulating non-pandemic strains, the impact of Gn pigs in HuNoV research cannot be understated. As the only animal model to develop diarrhea, shed virus, and seroconvert after oral inoculation, Gn pigs can be used to answer a breadth of research questions. One of the remaining open questions is how bacteria impact HuNoV infection. Moving forward, researchers can colonize Gn pigs with specific bacteria of interest, thereby determining how individual bacterial families influence HuNoV infection. These results can then be applied to humans, for example, by recommending probiotics containing the protective bacterial families for use as HuNoV prevention. The Gn pig models can be used to evaluate how unique immunocompromised states impact HuNoV infection. While simvastatin treatment universally increased susceptibility, two SCID phenotypes had different outcomes on infection. Using these study results, doctors can identify high-risk patients and communicate them potential risks. HuNoV vaccine studies in Gn pigs also have important implications for human health. No vaccine that fails in Gn pigs should progress to Phase I human clinical trials. Vaccine performance in Gn pigs is therefore a key step to progressing to clinical trials. In summary, this model has been invaluable and will continue to be an important tool for advancing the HuNoV research field in years to come.

## Figures and Tables

**Table 1:** Summary of HuNoV virus shedding and diarrhea in Gn pigs.

Name	Genotype	Dosage Used (Genome Copies)	Age Tested in Pigs	% Shedding Virus	Shedding Duration	Shedding Peak Titer (Genome Copies/mL) *	% with Diarrhea	Diarrhea Duration	Significance and Implications	Source
HS66	GII.4 Bristol	P0 **, 2.5 × 10^6^	5–7 days	73% (8/11)	3 days	NR	64% (7/11)	2 days	For the first time, Gn pigs are shown to be susceptible to HuNoV infection and display the range of clinical symptoms. Infectivity of HS66 maintained after two passages in Gn pigs.	Cheetham et al. [12]
		P1, 5 × 10^2^		29% (2/7)	2 days		86% (6/7)	3 days		
		P2, 4 × 10^1^		50% (2/4)	2 days		75% (3/4)	1 days		
		5.4 × 10^6^	5–7 days	91% (32/35)	4 days	NR	84% (27/32)	4	Higher doses of HuNoV inoculum increased severity of infection and clinical signs in Gn pigs.	Souza and Cheetham et al. [13]
		5.4 × 10^6^	33 days	57% (4/7)	NR	NR	100% (7/7)	NR	HS66 HuNoV can infect month-old Gn pigs.	Souza and Constantini et al. [14]
Cin2	GII.4 Farmington Hills	2 × 10^6^	33–34 days	100% (4/4)	2.5 days	6.94 × 10^3^	100% (4/4)	5 days	The ID_50_ of Cin2 in month-old pigs is 2.51 × 10^3^ RNA copies. This is 25.6-fold lower than GII.4 2006b (6.43 × 10^4^), indicating Cin2 is more infectious in Gn pigs.	Ramesh et al. [15]
		4 × 10^5^		100% (4/4)	1.3 days	4.57 × 10^3^	75% (3/4)	1.3 days		
		2 × 10^5^		100% (4/4)	6.3 days	4.44 × 10^4^	100% (4/4)	4 days		
		8 × 10^4^		100% (6/6)	2.8 days	1.16 × 10^4^	100% (6/6)	3.8 days		
		2 × 10^4^		67% (2/3)	1 day	8.7 × 10^1^	0% (0/3)	0 days		
		3.2 × 10^3^		67% (2/3)	1.3 days	9.51 × 10^3^	33% (1/3)	1 day		
		8 × 10^2^		25% (1/4)	0.5 days	2.25 × 10^3^	0% (0/4)	0 days		
2006b	GII.4 Den Haag	2.74 × 10^3^	4–5 days	50% (3/6)	11% of days ***	4.34 × 10^1^	67% (4/6)	32% of days ***	Neonates are more susceptible to GII.4/2006b infection than month-old pigs, as indicated by a lower ID50 in the neonates (<2.73 × 10^3^). Treatment of month-old pigs with simvastatin causes the ID50 to decrease, indicating immunosuppressed pigs are also more susceptible to HuNoV infection.	Bui et al. [16]
		2.74 × 10^5^		100% (3/3)	42% of days	1.09 × 10^4^	100% (3/3)	29% of days		
		2.74 × 10^3^	33–34 days	25% (1/4)	6.3% of days	1.29 × 10^1^	25% (1/4)	NR		
		2.74 × 10^4^		40% (2/5)	9% of days	5.47 × 10^1^	0% (0/5)			
		2.74 × 10^5^		67% (2/3)	11% of days	3.31 × 10^2^	0% (0/3)			
		2.74 × 10^6^		100% (3/3)	75% of days	4.31 × 10^4^	67% (2/3)			
HS194	GII.4[P4] Den Haag	2.4 × 10^9^ or 3 × 10^10^	11–13 days	NR	8.8 days	6.17 × 10^4^	0% (0/10)	0	HS194 infection presents asymptomatically in Gn pigs despite inoculation with a very high dose of virus and prolonged fecal virus shedding.	Jung et al. [17]
HS292	GII.4 New Orleans	1.26 × 10^9^	8–14 days	NR	11.6 days	1.58 × 10^6^	NR	NR	Based on higher peak titers of virus shedding in pigs, HS292 appears to be more infectious than HS194. However, it is not reported whether HS292 presents asymptomatically like HS194 or not.	Annamalai et al. [18]
KU131206	GII.4	1 × 10^5^	4 weeks	50% (2/4)	NR	NR	0% (0/4)	NR	Fecal KU131206 shedding is independent of dosage; however, diarrhea from KU131206 infection is reliant on dosage.	Park et al. [19]
		1 × 10^6^		75% (3/4)			25% (1/4)			
		1 × 10^7^		50% (2/4)			75% (3/4)			
GII.4 Sydney	GII.4 Sydney[P16]	2 × 10^5^	5 or 33 days	81% (9/11)	3 days	8.22 × 10^3^	64% (7/11)	3.83 days	GII.4 Sydney is moderately infectious in two different age groups of pigs. However, as these are pilot studies, future work to elucidate the exact ID_50_ and DD_50_ is necessary.	Yuan Lab, unpublished
GII.6	GII.6[P7]	5 × 10^4^	5 days	100% (2/2)	8.5 days	2.08 × 10^5^	50% (1/2)	2 days	GII.6 is infectious in neonates and month-old pigs. However, as these are pilot studies, future work to elucidate the exact ID50 and DD50 is necessary.	Yuan Lab, unpublished
		1.5 × 10^6^	33 days	100% (4/4)	5.75 days	6.37 × 10^4^	100% (4/4)	8.5 days		
HS206	GII.12[P33]	3.16 × 10^9^	8–11 days	100% (6/6)	7 days	1 × 10^6^	33% (2/6)	1.8 days	GII.12 is highly infectious in Gn pigs as evidenced by the high incidence and long duration of fecal shedding; however, the associated diarrhea incidence is low and the duration is short.	Jung et al. [20]
Recom GII.12 GII.3	GII.3[P12]	1 × 10^7^	4 weeks	100% (10/10)	2.4 days	NR	NR	NR	Recom-GII.12-GII.3-infected pigs had a short duration of virus shedding. Further work is needed to identify the diarrhea presentation and confirm replication of the virus as opposed to flow through.	Park et al. [21]

NR: not reported. * Virus titers measured by RT-qPCR and expressed as RNA copies/mL, genome equivalents/mL, and genome copies/g by different researchers are unified as genome copies/mL in this table for easier comparison. ** P0 is human stool filtrate; P1 and P2 are serial passaged intestinal contents of infected Gn pigs. *** The duration of diarrhea is presented as a percentage rather than number of days due to the euthanasia of piglets at multiple study timepoints.

**Table 2:** Summary of the ID50 and DD50 of HuNoV variants in Gn pigs.

Name	Variant	Age Tested in Pigs	Methods for Calculating ID50 and DD50	ID50 (Genome Copies)	DD50 (Genome Copies)	Source
Cin2	GII.4 Farmington Hills	33–34 days	Reed–Muench	2.51 × 10^3^	3.8 × 10^4^	Ramesh et al. [15]
			Dragstedt–Behrens	2.45 × 10^3^	3.8 × 10^4^	
			Spearman–Karber	3.31 × 10^3^	3.09 × 10^4^	
			Logistic Regression	2.51 × 10^3^	2.18 × 10^4^	
			Exponential	5.75 × 10^3^	5.75 × 10^4^	
			Approximate Beta-Poisson	2.57 × 10^3^	2.13 × 10^4^	
2006b	GII.4 Den Haag	4–5 days	Reed–Muench	2.74 × 10^3^	NR	Bui et al. [16]
		33–34 days		<2.74 × 10^3^ *		

NR: not reported. * 75% of pigs were infected at this dose; so, the ID50 is lower than the listed dose.

**Table 3:** Impact of host factors, gut microbes, drug treatments and immunocompromised health status on HuNoV infection in Gn pigs.

Name	Genotype	Condition/Treatment/Factor	Results	Source
HS66	GII.4 Bristol	Histo-blood group antigen expression	A+H+ increases virus shedding and diarrhea relative to A-H-	Cheetham et.al. [29]
766	GII.4	*Lactococcus lactis* (LAB) with or without expressing HuNoV VP1	All LAB-colonized pigs shedding similar titers of HuNoV in feces	Craig et al. [34]
2006b	GII.4 Den Haag	Pre-colonization with *Enterobacter cloacae* (*E. cloacae*)	*E. cloacae* decreases cumulative virus shedding and shedding duration	Lei et al. [35]
2006b	GII.4 Den Haag	Pre-colonization with human gut microbiome (HGM) sample	HGM increases duration of virus shedding and diarrhea	Lei et al. [36]
HS206	GII.12[P33]	Pre-colonization with *Escherichia fergusonii* (*E. fergusonii*)	*E. fergusonii* increases virus shedding duration and cumulative virus shedding	Jung et al., 2023 [26]
GII.4 Sydney	GII.4 Sydney[P16]	Pre-colonization with *E. fergusonii*	*E. fergusonii* increased diarrhea severity but did not significantly increase peak shedding titers	Yuan Lab, unpublished
Cin2	GII.4 Farmington Hills	Pre-colonization with *E. fergusonii*	*E. fergusonii* increased diarrhea severity but did not significantly increase peak shedding titers	Yuan Lab, unpublished
2006b	GII.4 Den Haag	Simvastatin	Increased incidence of diarrhea and virus shedding, longer virus shedding and higher peak titers in simvastatin-treated animals	Bui et al. [16]
2006b	GII.4 Den Haag	Simvastatin	Increased incidence of diarrhea and virus shedding and abolished the partial protective immunity induced by HuNoV P particle vaccine	Kocher et al. [37]
HS194	GII.4 Den Haag	Simvastatin	Greater duration and high peak HuNoV titers in simvastatin-treated animals	Jung et al. [17]
HS292	GII.4 New Orleans	Severe combined immunodeficiency (SCID) phenotype, T-B-NK+	No change in incidence of diarrhea or virus shedding when SCID phenotype was used	Annamalai et al. [18]
2006b	GII.4 Den Haag	RAG2/IL2RG gene knock-out with SCID phenotype, T-B-NK-	RAG2-/IL2RG- pigs had increased and prolonged viral shedding	Lei et al. [38]

## Data Availability

All relevant data are included in this manuscript.
